# Supplementary material for: Unveiling Fungi Armor: Preliminary Study on Fortifying Pisum sativum L. Seeds against Drought with Schizophyllum commune Fries 1815 Polysaccharide Fractions
Source: Microorganisms. 2024 May 29;12(6):1107. doi: 10.3390/microorganisms12061107 (PMC11205620; doi:10.3390/microorganisms12061107)
Supplement: Supplementary file 1 [file microorganisms-12-01107-s001.zip › microorganisms-2991774-supplementary.pdf]

	GE	SG	AS	SL	RL	SFW	RFW	SDW	RDW	SER	RER	SVI	MSI	EL	RWC	DTI	R/S ratio	SLSI	RLSI
GE	1,00																		
SG	0,54	1,00																	
AS	-0,26	-0,46	1,00																
SL	0,00	0,18	-0,42	1,00															
RL	-0,04	0,08	-0,18	0,19	1,00														
SFW	-0,12	0,18	-0,22	0,56	-0,21	1,00													
RFW	-0,08	0,24	-0,04	0,47	0,41	0,45	1,00												
SDW	0,03	0,14	-0,24	0,54	-0,09	0,85	0,38	1,00											
RDW	-0,06	-0,04	0,33	-0,11	0,30	-0,19	0,01	-0,28	1,00										
SER	-0,25	0,03	-0,12	0,66	0,24	0,03	0,16	0,04	0,02	1,00									
RER	-0,30	-0,09	0,06	-0,11	0,88	-0,28	0,34	-0,22	0,27	0,10	1,00								
SVI	0,07	0,31	-0,37	0,49	0,93	0,02	0,54	0,11	0,21	0,40	0,71	1,00							
MSI	0,28	0,14	-0,05	0,19	0,27	-0,10	0,40	-0,05	-0,28	0,03	0,17	0,31	1,00						
EL	-0,28	-0,14	0,05	-0,19	-0,27	0,10	-0,40	0,05	0,28	-0,03	-0,17	-0,31	-1,00	1,00					
RWC	0,21	0,21	-0,16	-0,65	0,08	-0,54	-0,46	-0,44	-0,12	-0,37	0,22	-0,08	0,05	-0,05	1,00				
DTI	-0,02	0,11	-0,06	0,54	0,11	0,80	0,44	0,87	0,20	0,09	-0,03	0,28	-0,14	0,14	-0,57	1,00			
R/S ratio	-0,51	0,02	0,36	-0,11	0,07	0,19	0,32	0,07	-0,04	0,21	0,34	0,04	0,01	-0,01	-0,08	0,12	1,00		
SLSI	-0,08	0,05	-0,25	0,81	-0,18	0,80	0,32	0,67	-0,12	0,32	-0,41	0,09	0,05	-0,05	-0,69	0,66	-0,05	1,00	
RLSI	0,15	0,48	-0,41	0,11	0,60	0,16	0,34	0,13	-0,08	-0,07	0,48	0,64	0,27	-0,27	0,27	0,14	0,01	-0,01	1,00

Figure S1. Heat map based on correlation analysis of investigated parameters of vegetable peas inoptimal conditions.GE – germination energy; SG – seed germination; AS – abnormal shoots; SL – shoot length; RL – root length; SFW – shoot fresh weight; RFW – root fresh weight; SDW – shoot dry weight; RDW – root dry weight; SER – shoot elongation rate; RER – root elongation rate; SVI – seedling vigor index; MSI – membrane stability index; EL – electrolyte leakage; RWC – relative water content; DTI – drought tolerance index; R/S ratio – root/shoot ratio; SLSI –shoot length tolerance index, RLSI – root length tolerance index.

	GE	SG	AS	SL	RL	SFW	RFW	SDW	RDW	SER	RER	SVI	MSI	EL	RWC	DTI	R/S ratio	SLSI	RLSI	SLSI	RLSI
GE	1,00																				
SG	0,80	1,00																			
AS	-0,62	-0,62	1,00																		
SL	0,45	0,57	-0,54	0,03	0,43	1,00															
RL	0,57	0,60	-0,55	0,10	0,58	0,90	1,00														
SFW	0,39	0,34	-0,22	0,26	0,63	0,56	0,78	1,00													
RFW	0,74	0,73	-0,41	0,63	0,56	0,22	0,26	0,15	1,00												
SDW	0,43	0,48	-0,10	0,27	0,40	0,43	0,67	0,76	0,26	1,00											
RDW	0,78	0,76	-0,41	0,51	0,58	0,40	0,53	0,56	0,61	0,68	1,00										
SER	0,13	0,28	-0,41	-0,44	0,07	0,88	0,76	0,38	-0,09	0,26	0,12	1,00									
RER	0,37	0,46	-0,49	-0,15	0,30	0,89	0,95	0,67	0,08	0,63	0,40	0,87	1,00								
SVI	0,62	0,71	-0,61	0,15	0,60	0,93	0,98	0,71	0,35	0,64	0,58	0,76	0,92	1,00							
MSI	0,37	0,26	-0,11	0,33	0,46	0,06	0,07	0,05	0,20	-0,03	0,17	-0,10	-0,10	0,10	1,00						
EL	-0,37	-0,26	0,11	-0,33	-0,46	-0,06	-0,07	-0,05	-0,20	0,03	-0,17	0,10	0,10	-0,10	-1,00	1,00					
RWC	0,28	-0,02	-0,36	-0,27	0,26	0,18	0,26	0,18	-0,12	-0,13	0,04	0,29	0,21	0,20	0,12	-0,12	1,00				
DTI	-0,04	-0,18	0,58	0,45	0,16	-0,35	-0,26	0,02	0,11	0,04	-0,06	-0,53	-0,37	-0,30	-0,11	0,11	-0,26	1,00			
R/S ratio	0,16	0,05	0,25	0,44	-0,13	-0,54	-0,56	-0,54	0,46	-0,18	0,11	-0,69	-0,61	-0,50	0,03	-0,03	-0,37	0,36	1,00		
SLSI	0,59	0,52	-0,52	0,37	0,45	0,71	0,62	0,27	0,46	0,24	0,42	0,47	0,55	0,67	0,25	-0,25	-0,05	-0,19	-0,03	1,00	
RLSI	0,73	0,54	-0,41	0,61	0,48	0,32	0,37	0,14	0,71	0,26	0,56	0,00	0,24	0,41	0,31	-0,31	-0,09	0,05	0,43	0,80	1,00

Figure S2. Heat map based on correlation analysis of investigated parameters of vegetable peas under drought. GE – germination energy; SG – seed germination; AS– abnormal shoots; SL – shoot length; RL – root length; SFW – shoot fresh weight; RFW – root fresh weight; SDW – shoot dry weight; RDW – root dry weight; SER – shoot elongation rate; RER – root elongation rate; SVI – seedling vigor index; MSI – membrane stability index; EL – electrolyte leakage; RWC – relative water content; DTI – drought tolerance index; R/S ratio – root/shoot ratio; SLSI –shoot length tolerance index, RLSI – root length tolerance index.
